# Genetic differentiation and historical dynamics of the endemic species *Rheum pumilum* on the Qinghai-Tibetan Plateau inferred from phylogeography implications

**DOI:** 10.1186/s12870-025-06164-y

**Published:** 2025-02-07

**Authors:** Shuliang Liu, Jianxin Gao, Bo Xiao, Wenjie Guo, Qiushi Yu, Ailan Wang, Weiwei Li

**Affiliations:** 1 School of Life Sciences, Ludong University, Yantai, Shandong 264025 China; 2 Gansu Academy of Forestry, Lanzhou, Gansu 730070 China

**Keywords:** *Rheum pumilum*, Phylogeography, cpDNA, The Qinghai-Tibetan Plateau, Ecological niche

## Abstract

**Background:**

*Rheum pumilum*, an endemic species on the Qinghai-Tibetan Plateau (QTP), serves as an ideal material for investigating the phylogeography of alpine plants. This study employs chloroplast DNA fragments (*trn*L-F, *trn*S-G, and *mat*K) to delve into how *Rh. pumilum* adapted to the extreme environmental changes on the QTP, during its evolutionary process through phylogenetic geographical analysis, revealing its population differentiation and historical dynamics.

**Results:**

The examination of 39 haplotypes across 26 populations of *Rh. pumilum* reveals distinct regional distribution, reflecting a phylogeographic pattern resembling “alpine-island”. The total genetic diversity of *Rh. pumilum* is remarkably high (Ht = 0.910), with the majority of genetic variation primarily occurred among populations (84.5%) with limited gene flow, indicating geographic isolation influenced by diverse habitats of plateau. The geographic isolation model is further supported by various analytical methods, including AMOVA analysis, UPGMA dendrogram, PCoA, Structure analysis, and Mantel test. Micro-refugia for *Rh. pumilum* during the Quaternary ice ages are supported by haplotype network and genetic diversity analysis. The absence of a typical “star-shape” pattern in the overall haplotype network suggests that *Rh. pumilum* likely maintains a stable state without experiencing rapid expansion, which has been supported by mismatch distribution analysis. Ecological Niche Modeling (ENM) indicates sensitivity of *Rh. pumilum* to humidity, temperature and altitude, aligning with a historical distribution resembling a “displacement refugia” model during the Quaternary ice ages. The involvement of *Rh. kialense* and *Rh. sublanceolatum* in the origin and gene introgression of *Rh. pumilum* is suggested, possibly as maternal ancestors of closely related haplotypes. Haplotype divergence of *Rh. pumilum* approximately 11 million years ago, with notable divergence peaks observed during the late Miocene, as well as the Pliocene, Pleistocene and Holocene.

**Conclusion:**

These findings suggest a correlation between genetic diversity, haplotype lineage divergence and key geological and climatic events, notably the uplift of the QTP, monsoon climate changes, and the climatic oscillations during the Quaternary ice ages. This study might provide valuable insights into the formation mechanisms of plant diversity on the QTP, crucial for biodiversity conservation and sustainable species development in extreme environments.

**Supplementary Information:**

The online version contains supplementary material available at 10.1186/s12870-025-06164-y.

## Introduction

The Qinghai-Tibetan Plateau (QTP) stands as the world’s highest and youngest plateau, characterized by extreme high-altitude conditions, aridity, cold temperatures, and intense ultraviolet radiation [1]. Its unique ecological environment has shaped an extraordinarily diverse alpine plant community, making it one of the global biodiversity hotspots [2–5]. The rapid uplift of the QTP during the late Tertiary period promoted population differentiation and radiative speciation, and the subsequent Quaternary ice ages accelerated the process of species differentiation and gave rise to very abundant alpine species [6–10]. The oscillating climate during the Quaternary glacial and interglacial periods had a profound impact on the distribution and genetic structure of plant species on the QTP [11–14]. These plants retreated to refuges during glacial periods, where conditions were more favorable for survival, and then expanded again during interglacial periods or after the last glacial period [13, 15–17]. Notably, the QTP experienced different influences from Europe and North America during the last glacial maximum (LGM), and did not form a unified large ice sheet [18], the plants in this region responded to the Quaternary glacial period with diversified phylogeographical patterns [19, 20]. Two main models of plant refuge have been proposed in the context of the QTP. The first model suggests that species retreated to the edges of the plateau along with the ice sheet, such as the edge of the eastern plateau or the lower elevations in the southeast, and then expanded to the plateau platform during the interglacial period [13, 16, 17, 21]. However, due to the founder effect and bottleneck effect during the interglacial and post-glacial periods, only a few haplotypes remained on the plateau. This resulted in more homogeneous and less genetically diverse populations, while populations at lower elevations on the plateau retained higher genetic diversity and more haplotypes [13, 16, 17, 21]. The second model suggests that during the Ice Age, as the species retreated, not all populations migrated to the low-altitude areas bordering the plateau; some populations remained in micro-refugia on the plateau platform [12, 22–26]. As the climate warmed during the interglacial and post-glacial periods, these populations expanded locally into surrounding areas, contributing to higher genetic diversity and more haplotypes in certain plateau populations [26, 27]. Research conducted in the QTP and its environs has identified adherence to these two models across various plant species [13, 28]. In addition, alpine plants have been reported to adopt other models to cope with the Quaternary glacial period. Recently, a novel “displacement refugia” model has been proposed to explain the distribution dynamics of plants in high-altitude regions, such as the European Alps and the Tibetan Plateau, during glacial and interglacial periods [29–33]. This model diverges from the traditional “contraction during glaciation, expansion post-glaciation” paradigm proposed by Hewitt [15]. The “displacement refugia” theory suggests that some alpine plants may have experienced range expansions during glacial period, followed by population contractions during interglacial or post-glacial times. The model is based on the premise that during glacial periods, the lowering of elevation may have led to a more contiguous distribution of alpine plants, thereby expanding their habitat range [15]. Furthermore, the extended duration of glacial periods relative to interglacial intervals [34, 35] would have provided more opportunities for population expansion, particularly for species adapted to cold environments. Conversely, during interglacial or post-glacial periods, an increase in habitat elevation resulted in geographic isolation and habitat fragmentation, leading to a contraction of the population’s distribution range [18, 36]. Hence, the degree of genetic differentiation among high-altitude plant populations is speculated to be lower than that observed in low-altitude plants. While some studies of plateau plants on the QTP support this hypothesis [36, 37], other researches have reported discrepancies. For example, studies of the alpine species *Eriophyton wallichii* (Labiatae) have revealed significant genetic divergence among populations [33]. This genetic divergence could be attributed to the extreme altitudes that *E. wallichii* inhabits on the Qinghai-Tibetan Plateau, where the decline in habitat elevation during glacial periods was negligible [33]. Therefore, populations remained isolated, and gene flow between them was limited. Beyond historical geological influences, factors like gene flow and genetic drift play pivotal roles in shaping genetic structures [38–40]. The intensity of gene flow directly influences population differentiation: larger gene flow results in reduced genetic divergence, and conversely, smaller gene flow leads to increased differentiation [41]. In general, as the geographic distance between populations increases, the opportunities for gene exchange diminish, amplifying the genetic differentiation [38, 39]. In relatively isolated small populations, restricted gene flow accentuates the impact of genetic drift [40]. Over multiple generations, this can result in the fixation of the same allele within the population and the genetic diversity of populations is diminished in instances of bottleneck or founder effects [42, 43]. The emergence of geographical patterns among plant lineages on the QTP is intricately linked to the plateau’s complex topography, geological history, and climatic conditions. Various plant groups on the plateau exhibit diverse and complex evolutionary histories [20]. In summary, to further understand the diversity mechanism, population history and systematic geographical pattern of alpine plants on the Tibetan Plateau, it is necessary to reconstruct the evolutionary history of a broader spectrum of plants inhabiting the plateau, especially the evolutionary history of typical endemic plants on the plateau.

*Rheum pumilum* Maxim. is a perennial herbaceous species of *Rheum* genus in the Polygonaceae family [44]. It lacks a distinct stem, which is a convergent evolutionary feature to adapt to extreme alpine environments (strong wind, drought, etc.) [6, 45]. This species is endemic to the QTP and is primarily distributed in high-altitude areas on the QTP and its neighboring regions, where it serves as a characteristic representative of the local flora [46]. Previous studies indicate that the *Rheum* genus has experienced a rapid radiative differentiation process [6, 47]. The uplift of the QTP, coupled with associated changes in habitat and climate, is proposed as the primary driving force behind the rapid radiative speciation within the *Rheum* genus [6, 45, 47]. Although QTP-related factors have been implicated in the origin and evolution of *Rh. pumilum*, it remains unclear how populations of this species have responded to the uplift and climatic changes of the QTP, as well as how they have adapted to the fluctuations between glacial and interglacial periods during the Quaternary. In a previous study [48], karyotype analysis revealed two distinct types of diploid and tetraploid in *Rh. pumilum*. Additionally, phylogenetic investigations in *Rheum* [6, 45, 47], utilizing chloroplast and nuclear gene fragments, highlighted a close relationship between *Rh. pumilum* and several other diploid species, hinting at a potential hybrid origin and the complex evolutionary history of *Rh. pumilum*, which necessitates further exploration. Investigating its mode of origin and geographical lineage can not only illuminate its evolutionary history but also provide valuable insights into the formation of genetic structures and distribution patterns of *Rh. pumilum* in relation to the unique geological history of the plateau.

In this study, we conducted a phylogeographic analysis of *Rh. pumilum* using three chloroplast DNA (cpDNA) fragments, aiming to address the following objectives: (1) Investigating the genetic diversity and structure of *Rh. pumilum* populations; (2) Estimating intraspecific lineage differentiation and identifying the maternal lineage of *Rh. pumilum*; (3) Inferring the refugia of *Rh. pumilum* and the population’s dynamics in response to the severe climatic fluctuations during the Quaternary glacial period. This study may provide further evidence for understanding the causes and mechanisms of plant diversification on the QTP, and it lays the groundwork for biodiversity conservation and the sustainable development of species in extreme environments.

## Materials and methods

### Samples collection

**Fig. 1:**
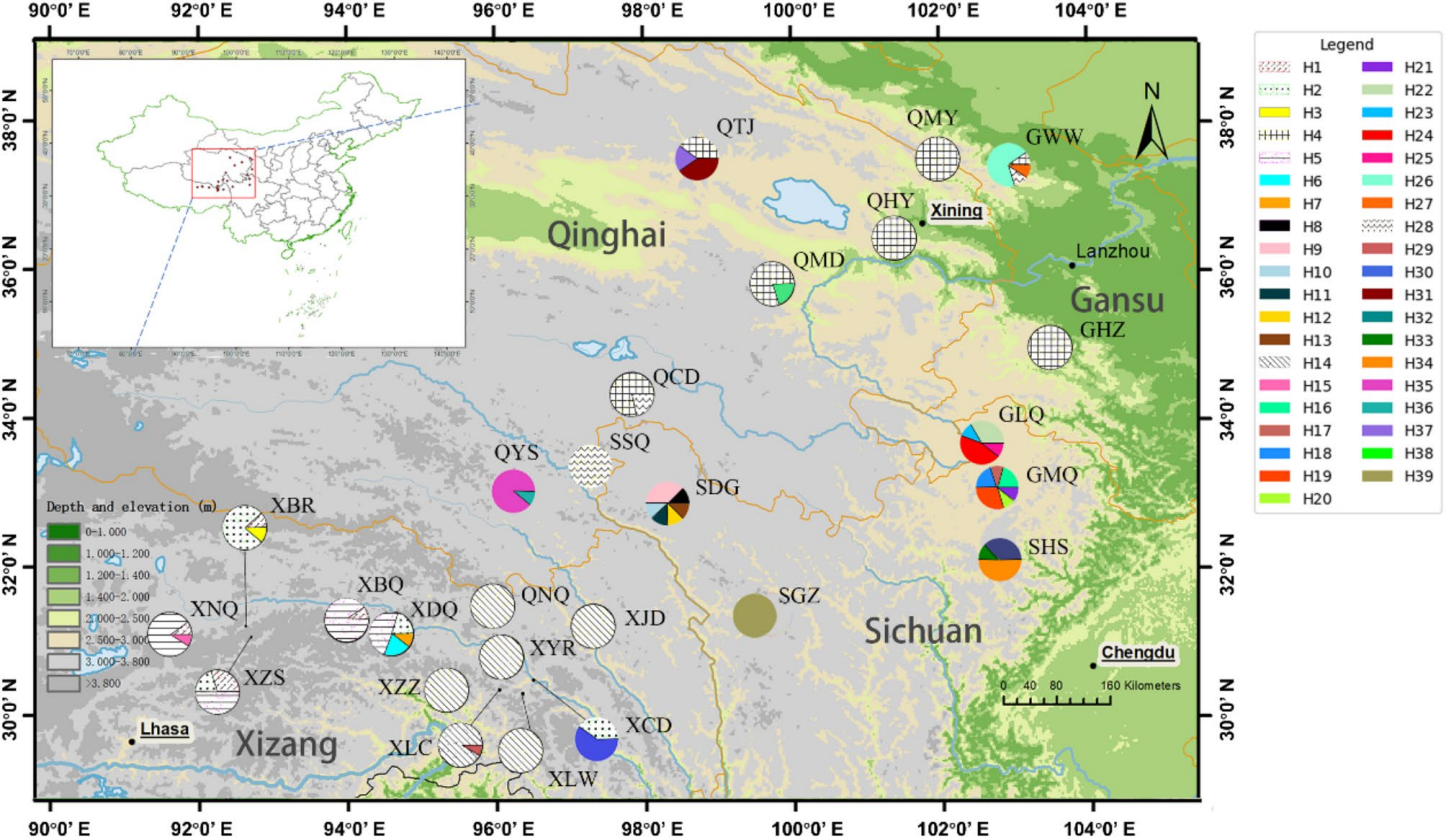
Distribution of 26 *Rh. pumilum* populations. In the pie chart, different colors and line patterns represent 39 different haplotypes, and the proportion of each color or pattern in the pie chart corresponds to the proportion of that haplotype in the population. A haplotype represented by a color is a unique haplotype of a population, while a haplotype represented by a line pattern is a shared haplotype among multiple populations

The distribution area of *Rh. pumilum* is mainly in Gansu, Qinghai, Xizang and Sichuan of the QTP and its adjacent areas, with elevations ranging from 2800 m to 4700 m. Twenty-seven representative populations were collected from the above areas, with distribution locations over a latitudinal range from 21.936° N to 35.8° N and a longitudinal range from 100.159° E to 119.767° E. These populations basically cover the main distribution area of *Rh. pumilum*. Except for one population with only 3 samples, which was discarded, the leaves of 240 individuals from the remaining 26 populations were collected and stored in silica gel for experimental analysis. To obtain the maximum genetic diversity within the population, samples collected from each population were spaced at least 5 m apart. The voucher specimens were morphologically identified by Ailan Wang from the School of Life Sciences, Ludong University (LDU), and deposited at the herbarium of LDU (voucher ID numbers: 2021008WA10-2021008WA35). The geographical distribution and altitude of the 26 populations are shown in Table 1; Fig. 1. Other species in *Rheum* for subsequent phylogenetic analysis are also included in Additional file 1: Table S1.

**Table 1:** Sampling locations and sample size in 26 populations of *Rh. Pumilum*

Populations	Location	Latitude	longitude	Altitude (m)	Sample size
GHZ	Hezuo, Gansu	35°06′59.73″	102°59′25.13″	3366	8
GLQ	Luqu, Gansu	33°59′52.37″	102°04′45.24″	3649	9
GMQ	Maqu, Gansu	33°15′24.68″	102°02′16.57″	3592	10
GWW	Wuwei, Gansu	37°15′40.49″	102°37′14.37″	3237	10
QCD	Chenduo, Qinghai	34°29′34.80″	97°59′08.52″	4400	9
QHY	Huangyuan, Qinghai	36°26′06.77″	101°07′13.82″	3427	9
QMD	Maduo, Qinghai	35°52′21.64″	99°41′00.33″	4255	10
QMY	Menyuan, Qinghai	37°19′48.70″	101°36′55.80″	2800	9
QNQ	Nangqian, Qinghai	31°56′13.73″	96°29′32.17″	4000	9
QTJ	Tianjun, Qinghai	37°20′30.00’’	98°48′10.02″	3800	10
QYS	Yushu, Qinghai	32°47′47.63″	96°38′17.44″	4160	9
SDG	Dege, Sichuan	32°56′17.95″	98°20′29.05″	4084	8
SGZ	Ganzi, Sichuan	31°51′02.94″	99°21′12.56″	3844	9
SHS	Heishui, Sichuan	32°12′31.40″	102°33′39.49″	3519	8
SSQ	Shiqu, Sichuan	33°8′02.04″	97°27′56.16″	4700	9
XBQ	Baqing, Xizang	31°50′06.72″	94°18′58.68″	4337	10
XBR	Biru, Xizang	31°52′03.11″	93°31′13.83″	4532	9
XCD	Changdu, Xizang	31°12′52.96″	96°53′54.96″	3871	5
XDQ	Dingqing, Xizang	31°40′12.01″	94°49′48.03″	4513	10
XJD	Changdu, Xizang	31°16′49.68″	97°33′40.27″	3881	8
XLC	Leiwuqi, Xizang	31°06′54.02″	96°30′02.66″	4338	12
XLW	Leiwuqi, Xizang	31°13′26.61″	96°36′01.08″	3885	10
XNQ	Naqu, Xizang	31°45′29.40″	92°37′32.13″	4560	11
XYR	Yinri, Xizang	31°33′26.80″	96°34′13.99″	4268	9
XZS	Suoxian, Xizang	31°45′30.96″	93°34′24.23″	4184	10
XZZ	Dingqing, Xizang	31°07′02.65″	95°51′35.86″	4426	10
Total					240

### Methods

#### DNA extraction and sequencing

Referring to the 9 chloroplast gene fragments used in the previous phylogenetic analysis of *Rheum* [6, 47, 49, 50], three gene fragments (*trn*L-F, *trn*S-G, and *mat*K) were selected with more variable sites for the phylogeographic analysis at the population level. Total DNA was extracted from silica gel-dried leaves materials collected from all regions using the CTAB method [51]. DNA concentration was detected using spectrophotometer and agarose gel electrophoresis and adjusted to 40 ng/µL for subsequent PCR reaction. The *trn*L-F sequences, *trn*S-G sequences, and *mat*K sequences were amplified using the primers of ‘F’ (5’- CGAAATCGGTAGACGCTACG-3’) and ‘R’ (5’- GATTTGAACTGGTGACACGAG − 3’) [53], ‘S’ (5’-GCCGCTTTAGTCCACTCAGC) and ‘G’ (5’-GAACGAATCACACTTTTACCAC) [54], and ‘XF’ (5’-TAATTTACGATCAATTCATTC) and ‘5R’ (5’-GTTCTAGCACAAGAAAGTCG) [54], respectively. The PCR reactions were performed in a 25 µL reaction system containing 40 ng template DNA, 12.5 µL of 2×Taq DNA polymerase PCR Master Mix buffer, 1 µL of each primer at 5 µM and 1µL of plant DNA and 9.5 µL of sterile double-distilled water. The PCR program was as follows: initial template denaturation at 94 ℃ for 5 min, followed by 38 cycles of 94 ℃ for 50 s, 52℃ (*mat*K, *trn*L-F) or 56℃ (*trn*S-G) for 50 s, and 72 ℃ for 1.25 min, with a final extension of 72 ℃ for 8 min. PCR products were examined by electrophoresis on 1.0% (w/v) agarose gel. Qualified products sequencing was completed at Beijing Genomics Institute (BGI). The sequences of 20 *Rheum* species and *Oxyria digyna* as an outgroup (Additional file 1: Table S1) were obtained from our earlier results of a large-scale phylogenetic analysis of *Rheum* and others species of sister genus [6, 45, 47].

#### Data analysis

All original sequences were aligned using CLUSTAL X [52] and MEGA7.0 software [53]. PAUP 4.0 software was used for sequence splicing and homogeneity testing [54]. The DnaSP 5.0 software [55] was used to determine sequence haplotypes, calculate and analyze the number and distribution of haplotypes, calculate haplotype diversity (Hd), nucleotide diversity (Pi) and assess gene flow between populations. The genetic diversity index (Ht and Hs) and genetic differentiation coefficient (G_ST_ and N_ST_) at the population level of *Rh. pumilum* were computed using PERMUT 2.0 software [56] with 1000 permutation tests for significance. Arlequin 3.5.2 [57] was employed for the Analysis of Molecular Variance (AMOVA), calculating the inter-population genetic differentiation index F_ST_, evaluating the level of genetic variation within and between populations, and analyzing the dominant factors influencing genetic differentiation. Based on the genetic distance between populations, a UPGMA dendrogram was constructed using Mega7.0 software [53]. The “ape” package [58] in R studio was used for genetic distance calculation and PcoA analysis, and the obtained data was plotted with the “ggplot2” package [59]. To assess the correlation between population genetic distance and geographic distance, a Mantel test [60] was conducted with 10,000 replicates. To assess whether the population of *Rh. pumilum* has undergone expansion, the mismatch distribution curve was computed using DnaSP 5.0 software [55]. Populations experiencing demographic expansion typically exhibit a unimodal mismatch distribution, while those maintaining a stable size display a multimodal mismatch distribution [61]. Neutral deviation detection was carried out through Tajima’s D and Fu’s Fs analyses using DnaSP 5.0 [55]. Arlequin 3.5.2 software [57] was employed to calculate the Sum of Squared Deviations (SSD) between observed and expected mismatch distributions and Harpending’s Rag index (HRag) to assess the smoothness of the mismatch distribution. Network software [62] was used to construct a haplotype network of *Rh. pumilum* through median joining networks. The phylogenetic relationships of *Rh. pumilum* lineages, combined with 20 other species in the *Rheum* genus using *Oxyria digyna* as an outgroup, were established. The BEAST analyses were performed based on two available fossil records [47, 63] to estimate the divergence times between haplotypes and species, employing a Relaxed Clock Exponential with a Birth Death model [64]. The Markov Chain Monte Carlo (MCMC) analysis was run for 100 million generations, with sampling every 1,000 generations and the first 50% samples were discarded as burn-in. Convergence of the MCMC runs was assessed using Tracer v.1.7.2. TreeAnnotator v.2.7.7 [64] was used to summarize the set of post-burn-in trees and their parameters, generating a maximum clade credibility chronogram that displays the mean divergence time estimates with 95% highest posterior density (HPD) intervals. Structure 2.3.4 [65] was employed for Bayesian clustering analysis. The K values were set from 2 to 26, and each K value was repeated 10 times to detect the optimal K value. The number of MCMC generations was set to 100,000. Cluster repeat sampling analysis was performed using CLUMMP [66].

The geographical distribution patterns of *Rh. pumilum* at different history stages were predicted based on the distribution data of *Rh. pumilum* samples and bioclimatic variables using Ecological Niche Modeling (ENM). Nineteen bioclimate environmental variables from all sample distribution locations in the future (2060–2080) (CMCC-ESM2), current, mid Holocene (CCSM4), and last glacial period (CCSM4), as well as one altitude variable in the current layer were selected. Due to the interference caused by the correlation among the initial 19 bioclimatic variables in partition reconstruction, a Pearson correlation analysis was conducted on the 19 climatic factors related to the distribution points of *Rh. pumilum* using the Spatial Analyst tool in ArcGIS 10.2. when the correlation between two variables exceeded 0.8, only the variable with clearer biological significance was selected for model construction. Ultimately, nine environmental variables were selected from a total of 20 for predicting the potential distribution area: bio1, bio2, bio4, bio5, bio10, bio11, bio13, bio15, and Elev (Additional file 2: Table S2). The MaxEnt 3.4.4 [67] was used to construct the suitable habitat model. The distribution point data and the nine environmental variable data were imported into MaxEnt 3.4.4, with 25% of the distribution points chosen as the test set and 75% as the training set for model development. The bootstrapping was repeated 10 times with 500 iterations and a convergence threshold of 0.00001, while keeping other parameters at their default settings. The distribution area of *Rh. pumilum* was divided into four levels according to the fitness value: non-suitable area (fitness value < 0.1), low suitable area (0.1 ≤ fitness value < 0.3), medium suitable area (0.3 ≤ fitness value < 0.5), and high suitable area (fitness value ≥ 0.5), and the potential distribution area under the main climate scenarios in four different geological historical periods was statistically analyzed.

## Results

### Chloroplast sequence characteristics and haplotype polymorphism of *Rh. pumilum*

Chloroplast fragments (*trn*L-F, *trn*S-G, *mat*K) were sequenced and analyzed from 240 individuals across 26 populations of *Rh. pumilum*. After manual correction, *trn*L-F fragments with the length ranged from 843 bp to 867 bp, *trn*S-G fragments with the length ranged from 875 bp to 926 bp, and *mat*K fragments with a length of 842 bp were obtained. Following alignment with MEGA7 software, the combination of these three fragments yielded a nucleotide matrix spanning 2725 bp, encompassing 2479 conserved nucleotide sites. Additionally, there were gaps or missing data at 178 sites and 68 single nucleotide mutation sites, resulting in a mutation rate of approximately 10%. Among the 68 identified single nucleotide mutations, 61 were parsimony informative sites, and seven were singleton variable sites. The remaining mutations included long segment insertions or deletions, treated equivalently to single nucleotide mutations in subsequent analyses. The combined fragment presented 99 mutation sites, and detailed information on mutation bases is available in Additional file 3: Table S3. In total, 39 haplotypes were identified across the 26 populations of *Rh. pumilum* (GenBank accession numbers: PP049090 - PP049128 for *trn*L-F sequences, PP049129 - PP049167 for *mat*K sequences, PP049168 - PP049206 for *trn*S-G sequences). Their distribution in each population is outlined in Table 2; Fig. 1. Notably, the occurrence frequency of each haplotype varied significantly. The three most frequent haplotypes, namely H14, H4, and H5, were observed in 57, 46, and 28 individuals, respectively, constituting 23.75%, 19.17%, and 11.67% of the total. The haplotype H14 is predominantly found in populations XJD, XLC, XLW, XYR and XZZ from Xizang, and QNQ from Qinghai; Haplotype H4 is distributed in populations QCD, QHY, GHZ, QMD, QMY, and QTJ in Qinghai, and GWW from Gansu; Haplotype H5 is observed in populations XBQ, XDQ, XNQ, and XZS from Xizang; Haplotypes H1, H2, and H28 are prevalent in multiple populations. Interestingly, the remaining 33 haplotypes were found in only one population (Fig. 1), resembling a geographical distribution pattern of “alpine-island”. Additionally, four populations from Gansu exhibit 14 haplotypes (H4, H16-H28), three populations from Sichuan exhibit 11 haplotypes (H8-H13, H28, H32-H34, and H39), seven populations from Qinghai display 8 haplotypes (H4, H14, H28, H30-H31, H35-H36, H38), and 11 populations from Xizang have 10 haplotypes (H1-H3, H5-H7, H14-H15, H29, H37). The distribution of haplotypes was statistically analyzed by province, and the results revealed that only H4, H14, and H28 haplotypes are distributed in two provinces, indicating that the distribution of haplotypes is geographically restricted. In terms of a single population, excluding 10 populations with only one haplotype, the rest of 16 the populations exhibit multiple haplotypes (Table 2). Notably, GMQ from Gansu and SDG from Sichuan stand out as populations with the highest haplotype diversity, each containing six haplotypes. Following closely are XDQ from Xizang, GLQ and GWW from Gansu, each with four haplotypes. Additionally, five and six populations contain three and two haplotypes, respectively (Table 2). In summary, the distribution of haplotypes in *Rh. pumilum* exhibits characteristics of geographical differentiation. Although there are instances where a single population contains multiple haplotypes, the distribution areas of the main haplotypes do not overlap, demonstrating a pattern of north-south regional differentiation that is separated by latitude.

**Table 2:** CpDNA haplotype distribution and diversity in 26 populations of *Rh. pumilum* on the Qinghai-Tibetan Plateau

Population	Haplotype (sample numbers)	Number of haplotype	Haplotype diversity (Hd)	Nucleotide diversity (Pi)
GHZ	H4(8)	1	0.000 ± 0.000	0.00000 ± 0.00000
GLQ	H22(3), H23(1), H24(4), H25(1)	4	0.639 ± 0.126	0.00113 ± 0.00030
GMQ	H16(2), H17(1), H18(2), H19(3), H20(1), H21(1)	6	0.889 ± 0.080	0.00251 ± 0.00044
GWW	H27(1), H28(1), H26(7), H4(1)	4	0.756 ± 0.180	0.00102 ± 0.00026
QCD	H4(7), H28(2)	2	0.389 ± 0.164	0.00061 ± 0.00026
QHY	H4(9)	1	0.000 ± 0.000	0.00000 ± 0.00000
QMD	H4(8), H38(2)	3	0.356 ± 0.159	0.00110 ± 0.00049
QMY	H4(9)	1	0.000 ± 0.000	0.00009 ± 0.00000
QNQ	H14(9)	1	0.000 ± 0.000	0.00000 ± 0.00000
QTJ	H4(4), H30(2), H31(4)	3	0.711 ± 0.178	0.00034 ± 0.00018
QYS	H35(8), H36(1)	2	0.222 ± 0.166	0.00052 ± 0.00039
SDG	H8(1), H9(3), H10(1), H11(1), H12(1), H13(1)	6	0.893 ± 0.111	0.00345 ± 0.00142
SGZ	H39(9)	1	0.000 ± 0.000	0.00000 ± 0.00000
SHS	H32(3), H33(1), H34(4)	3	0.679 ± 0.122	0.00064 ± 0.00012
SSQ	H28(9)	1	0.000 ± 0.000	0.00022 ± 0.00000
XBQ	H1(1), H5(9)	2	0.200 ± 0.154	0.00023 ± 0.00018
XBR	H1(1), H2(7), H3(1)	3	0.417 ± 0.191	0.00069 ± 0.00040
XCD	H2(2), H37(3)	2	0.600 ± 0.175	0.00117 ± 0.00034
XDQ	H2(2), H5(5), H6(2), H7(1)	4	0.733 ± 0.120	0.00067 ± 0.00018
XJD	H14(8)	1	0.000 ± 0.000	0.00000 ± 0.00000
XLC	H14(11), H29(1)	2	0.167 ± 0.134	0.00013 ± 0.00011
XLW	H14(10)	1	0.000 ± 0.000	0.00000 ± 0.00000
XNQ	H1(1), H5(9), H15(1)	3	0.327 ± 0.153	0.00038 ± 0.00018
XYR	H14(9)	1	0.000 ± 0.000	0.00000 ± 0.00000
XZS	H1(3), H2(2), H5(5)	3	0.689 ± 0.104	0.00094 ± 0.00011
XZZ	H14(10)	1	0.000 ± 0.000	0.00000 ± 0.00000
Total	-	39	0.897 ± 0.013	0.00609 ± 0.0006

At the species level, the haplotype diversity (Hd) of *Rh. pumilum* is remarkably high at 0.897, accompanied by a relatively high nucleotide diversity (Pi) of 0.00609, indicating that *Rh. pumilum* has relatively high chloroplast gene sequence variation and high intraspecific genetic diversity. At the population level, eighteen populations showed haplotype polymorphism (Table 2), with the highest haplotype diversity population being SDG (Hd = 0.893 ± 0.111, Pi = 0.00345 ± 0.00142), distributed in the Hengduan Mountains in Sichuan, followed by GMQ (Hd = 0.889 ± 0.080, Pi = 0.00251 ± 0.00044), GWW (Hd = 0.756 ± 0.180, Pi = 0.00102 ± 0.00026), distributed on the edge of the plateau in Gansu, XDQ (Hd = 0.733 ± 0.120, Pi = 0.00067 ± 0.00018) and QTJ population (Hd = 0.711 ± 0.178, Pi = 0.00034 ± 0.00018), distributed on the platform of the QTP (Table 2). Notably, the XZS, SHS, GLQ and XCD populations also showed notably high population diversity (Hd = 0.600 − 0.689, Pi = 0.00117 − 0.00094). Furthermore, twelve populations exhibited high population diversity levels (Hd = 0.167–0.417, Pi = 0.00013–0.00069), while the remaining eight populations showcased low haplotype and nucleotide diversity levels (Hd = 0, Pi = 0) (Table 2).

### Genetic diversity and genetic structure analysis of *Rh. pumilum*

The genetic diversity index for the whole species of *Rh. pumilum* was computed using PERMUT software. The overall genetic diversity (Ht) of the population was found to be 0.910 (*P* = 0.0330), while the average genetic diversity within the population (Hs) was 0.330 (*P* = 0.0637), with the former significantly surpassing the latter. The genetic variation coefficient of populations, G_ST_= 0.638 (*P* = 0.0621), and N_ST_ = 0.897 (*P* = 0.0266), with N_ST_ > G_ST_, and the difference was statistically significant (*P* < 0.01). This suggests that the haplotypes of *Rh. pumilum* with close genetic distance also have short geographical intervals between each other, establishing a positive correlation between genetic differences and geographical distance. The notable genetic differentiation among populations implies a significant phylogeographical structure in the distribution of *Rh. pumilum*.

**Fig. 2:**
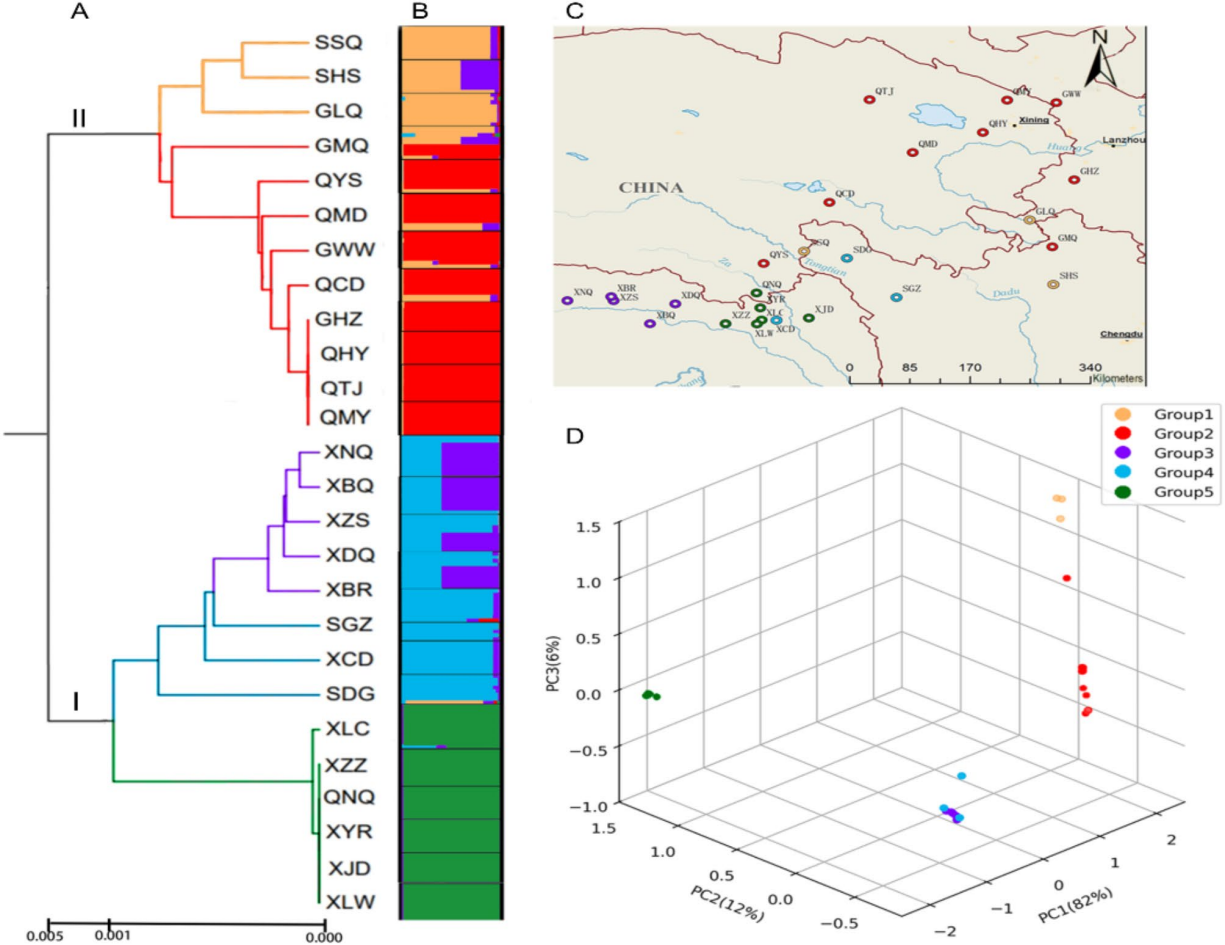
Genetic structure analysis of *Rh. pumilum populations.*** A** The UPGMA dendrogram of *Rh. pumilum* populations; **B** The cluster analysis of *Rh. pumilum* populations based on Structure software; **C** The locations of *Rh. pumilum* populations; **D** Principal Coordinate Analysis(PCoA) of *Rh. pumilum* populations. The obtained five groups were marked with different colors; I and II represent the two major branches in UPGMA tree

An UPGMA dendrogram was constructed based on genetic distance for the 26 populations. The results revealed a clear bifurcation into two clusters (I and II) (Fig. 2A). Cluster I comprised 14 populations, 11 from Xizang, one from Qinghai, and two from Sichuan. Among them, five populations (XLC, XZZ, XYR, XJD and XLW) from Xizang and the QNQ population from the neighboring province of Qinghai exhibited a closer genetic relationship (populations with close genetic relationships are represented by the same color in Fig. 2); five populations (XNQ, XBQ, XZS, XDQ and XBR) from Xizang gather into a subgroup, while two populations (SGZ and SDG) from Sichuan and one population (XCD) from Xizang show a closed relationship. Cluster II included 12 populations, six from Qinghai, four from the neighboring province of Gansu, and two from Sichuan. Within this cluster, the GLQ population from Gansu demonstrated a closer genetic relationship with the SSQ and SHS populations from Sichuan, while the remaining three populations from Gansu were more closely related to all six populations in Qinghai. The clustering analysis clearly indicated that populations located in close geographical proximity tended to cluster together, suggesting a direct correlation between geographical distance and genetic distance. To further explore the phylogeographic pattern of genetic variation in *Rh. pumilum* populations, the cluster analysis was performed using the Structure software. At K = 5, a distinct peak emerged (Additional file 1: Figure S1), delineating the population structure of *Rh. pumilum* into five segments (Fig. 2C). This result strongly supported the findings from the UPGMA dendrogram and PCoA analysis. Populations in close geographical locations, such as the GMQ, GWW populations from Gansu, and the QYS, QCD, QMD populations from Qinghai, exhibited genetic admixture. Similarly, the XCD populations from Xizang, and the SDG, SGZ populations from Sichuan also displayed intermixture. It is suggested that there may be gene flow among these populations and there is a closer genetic relationship. A Mantel test was employed to analyze the correlation between the average genetic distance and geographical distance of 26 *Rh. pumilum* populations. The results revealed a strong, significant positive correlation (*r* = 0.5522, *P* < 0.01), indicating that *Rh. pumilum* exhibits characteristics of isolation by distance, consistent with the geographic isolation hypothesis. These results were further supported by the PCoA analysis (Fig. 2D), where the first three axes accounted for 82%, 12%, and 6% of the total genetic variation, respectively. The populations were still successfully divided into five distinct groups corresponding to the UPGMA dendrogram and Structure analysis, although Groups 3 and 4 were genetically too similar to be easily distinguished.

To explore the genetic structure within and between *Rh. pumilum* populations, the AMOVA analysis was performed. The analysis identified significant genetic differentiation among *Rh. pumilum* populations (F_ST_ = 0.8450) (Additional file 4: Table S4), with 84.50% of genetic variation occurring between populations and 15.50% within populations. This suggests that genetic variation in *Rh. pumilum* predominantly occurs between populations. This outcome aligns with gene flow calculations (Nm = 0.04, Nei’s 1982), indicating minimal gene flow between populations. This suggests that diverse geographical environments on the QTP may have contributed to isolating *Rh. pumilum* populations.

### Historical evolutionary relationships and geographical distribution patterns among *Rh. pumilum* haplotypes

**Fig. 3:**
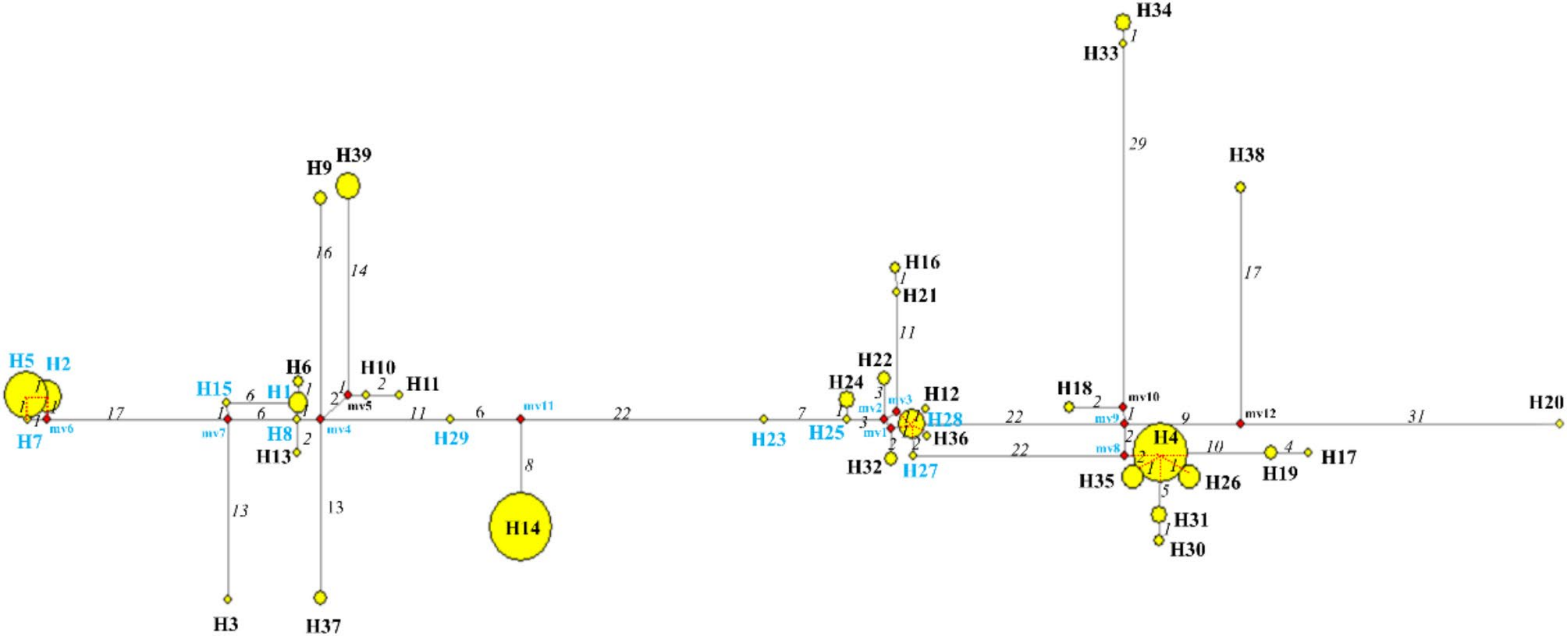
The network diagram of 39 haplotypes of *Rh. pumilum* based on NETWORK software. Each circle represents a haplotype, with the size of the circles is proportional to the frequency of haplotype occurrence; The mv1-mv12 represent “undetected haplotypes”; The haplotypes in blue font are core haplotypes, and the haplotypes in black font are the derived haplotypes; Italic numbers refer to the number of mutations between connected haplotypes; The red dashed line indicates connection lines obscured by the circle

**Fig. 4:**
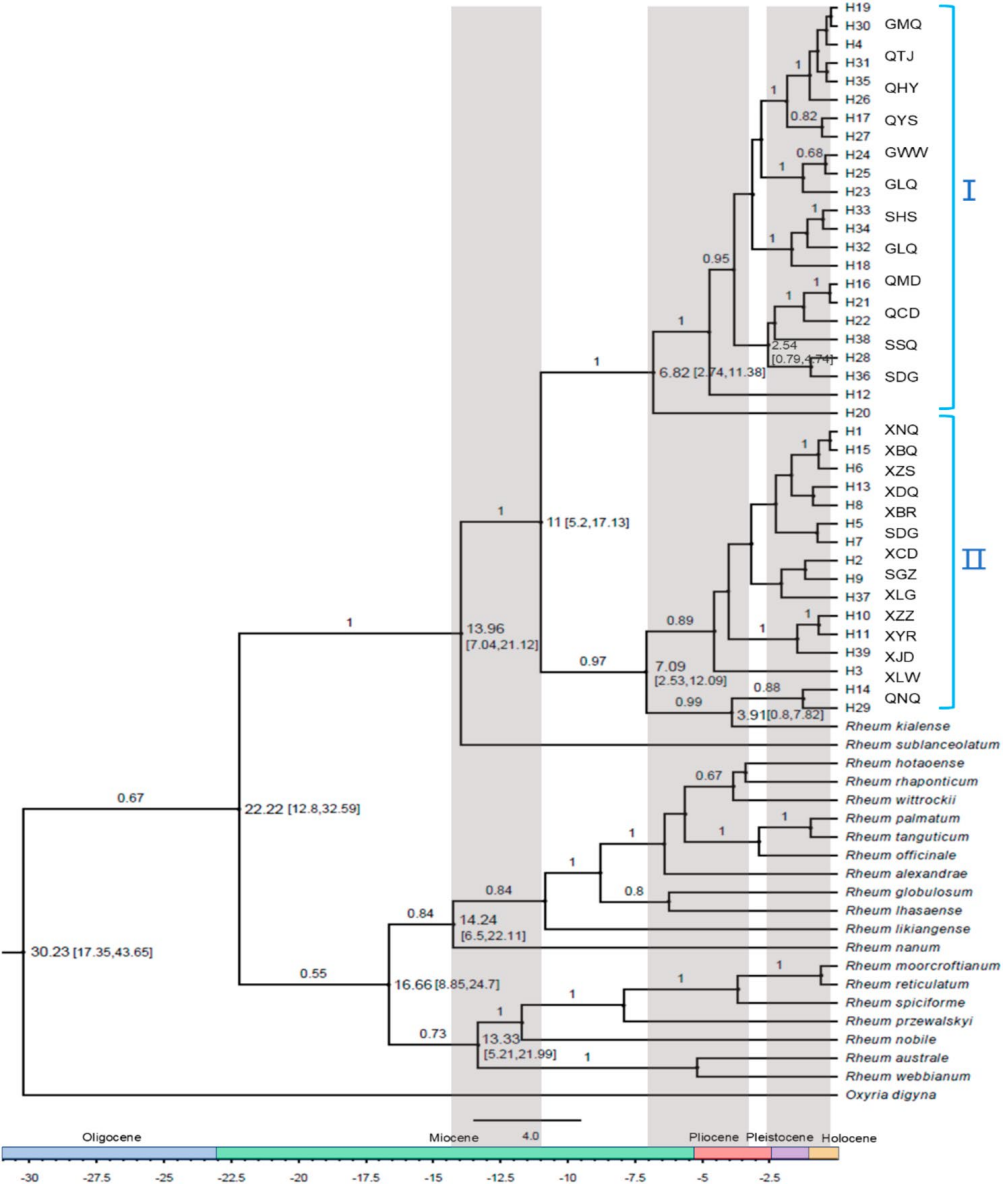
The phylogenetic tree constructed based on 39 haplotype sequences and the sequences of sister species of *Rh. pumilum* based on Bayesian method using BEAST software. *Oxyria digyna* was used as an outgroup. The divergence time, the 95% highest posterior density (HPD) interval (right side of the nodes), and posterior probabilities > 0.5 (above branches) are shown. For simplification, Group I and Group II were marked on the cpDNA tree, and the population number corresponding to the haplotype of the two groups are also marked on the tree. The gray background represents a period of rapid differentiation of *Rheum* species

Utilizing the NETWORK software, we constructed a network of 39 *Rh. pumilum* haplotypes employing the maximum parsimony median-joining method (Fig. 3). The proportional size of the circles in the figure represents the frequency of haplotype occurrence. It can be seen that haplotypes H1, H2, H5, H7, H8, H15, H23, H25, H27, H28 and H29, along with nine elusive “undetected haplotypes” (ancient haplotypes yet not to be detected or that may have become extinct), form the “torso” of the network; Other haplotypes are connected to these as derived haplotypes (Fig. 3). Considering the results of the analysis of haplotype divergence times (Fig. 4), the existing haplotypes cannot be definitively identified as ancestral haplotypes. Consequently, it is inferred that the ancestral haplotypes may be “undetected haplotypes”. The overall network configuration is elongated and linear, with considerable distances separating many haplotypes, indicative of a prolonged evolutionary history. Several haplotypes exhibit connections to multiple others, forming a complex “loop” structure. It could suggest the occurrence of gene convergence [68, 69] or introgression [70] in the evolutionary history of *Rh. pumilum*. Notably, the absence of a typical “star-shape” structure in the haplotype network suggests that *Rh. pumilum* likely did not experience a rapid expansion in the recent past. In addition, the 39 haplotypes were also clearly divided into two major groups based on their geographical distribution, as revealed by UPGMA analysis.

Based on 39 haplotype sequences, combined with data from 20 other previously reported species in the *Rheum* genus (Additional file 1: Table S1), a total of 61 sequences were aligned, resulting in a 2928 bp nucleic acid matrix. A phylogenetic tree was subsequently constructed using BEAST, with *Oxyria digyna* serving as the outgroup (Fig. 4). The relationships among haplotypes, as shown in the phylogenetic tree, align closely with the results of the NETWORK analysis, with high Bayesian posterior probabilities (BP) supporting the clades. The haplotypes form two distinct lineages: a monophyletic group I and a paraphyletic group II, as marked on the cpDNA tree (Fig. 4). The haplotypes H3, H4, H12, H16-H19, H20-H28, H30-H36, and H38 cluster together with robust support (BP = 100%). Similarly, haplotypes H1, H2, H5-H11, H13-H15, H29, H37, H39, and *Rh. kialense* form another cohesive group with 97% support. Notably, haplotypes H14 and H29 are sister taxa and together form a sister group with *Rh. kialense*, with support rates of 88% and 99%, respectively (Fig. 4). *Rh. sublanceolatum* clusters at the base of all *Rh. pumilum* haplotypes with strong support (100%), suggesting its potential role in the origin and gene introgression of *Rh. pumilum*.

Consistent with the two haplotype lineages in the phylogenetic tree and their corresponding population distribution, the 26 populations associated with these haplotypes were also divided into two groups (I and II, Fig. 4). This division closely matches the genetic relationships among populations, as illustrated by the topological structure derived from UPGMA analysis (Fig. 2A). Group I includes six populations from Qinghai (QYS, QCD, QHY, QMD, QMY, QTJ), four populations from the neighboring province of Gansu (GHZ, GLQ, GMQ, GWW), and two populations from Sichuan (SSQ, SHS). Group II includes one population from Ningxia (QNQ), two populations from Sichuan (SDG, SGZ), and all 11 populations from Xizang (XBQ, XBR, XCD, XDQ, XJD, XLC, XLW, XNQ, XZS, XZZ, XYR). Interestingly, when analyzing the population groups corresponding to these haplotypes, we found that one haplotype from the SDG population was placed in Group I, while the remaining haplotypes were found in Group II. This may be due to the SDG population’s location in a transitional area between the two groups, facilitating gene flow between populations from Qinghai and Gansu. Consequently, the haplotype evolutionary tree continues to support the genetic relationship between haplotypes in the two principal distribution areas of *Rh. pumilum*, forming two major lineage groups.

### Historical dynamic analysis of *Rh. pumilum* populations

**Fig. 5:**
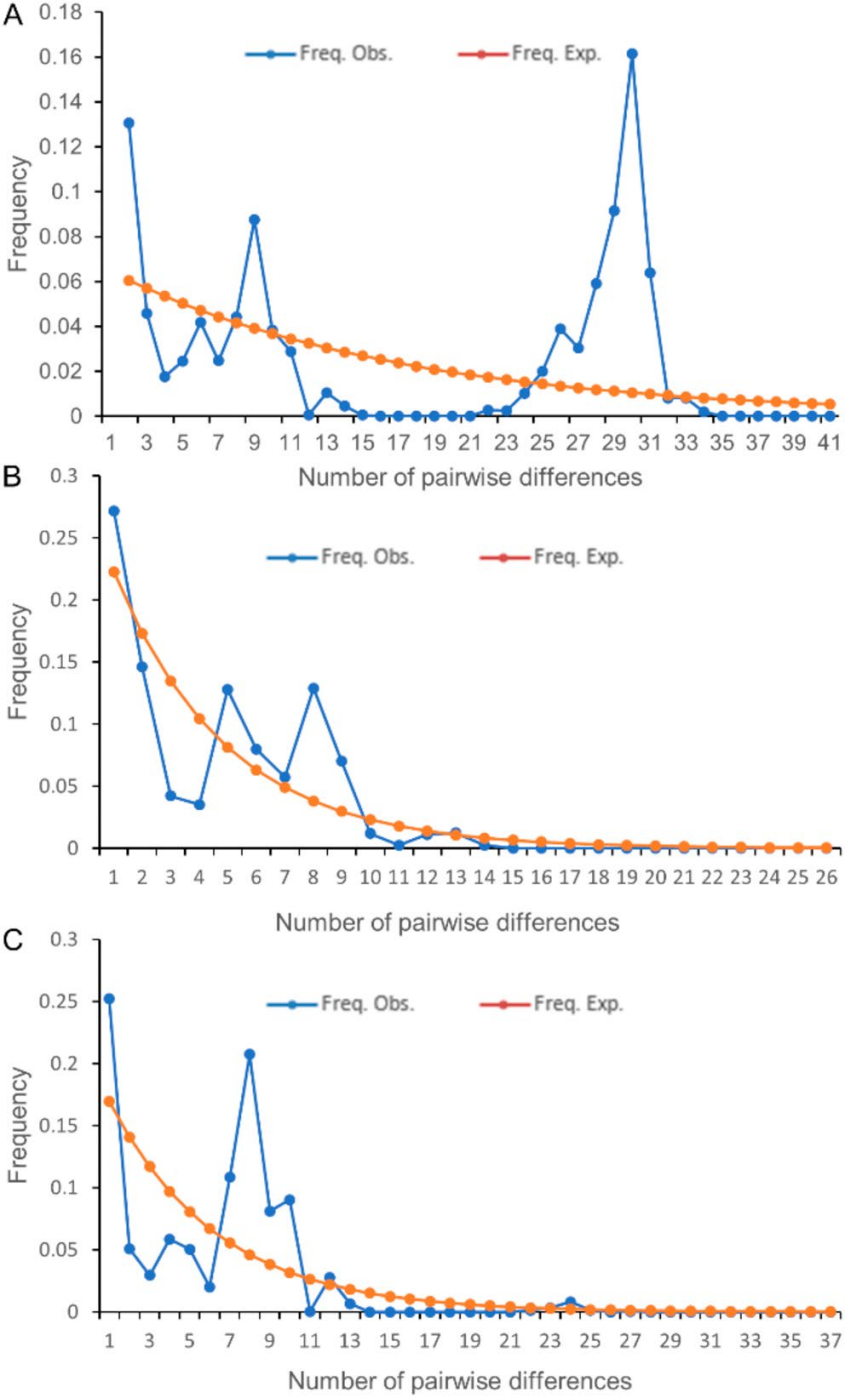
The mismatch distribution analysis of *Rh. pumilum* populations. **A** The mismatch distribution curve of all the *Rh. pumilum* populations; **B** The mismatch distribution curve of *Rh. pumilum* populations from Qinghai; **C** The mismatch distribution curve of *Rh. pumilum* populations from Xizang

To elucidate the population dynamics of *Rh. pumilum* during its evolutionary process, we employed a mismatch distribution analysis utilizing DnaSP 5.0 software [55]. The results revealed a distinctive double-peak mismatch distribution curve (Fig. 5A), indicating the absence of expansion events and suggesting that the population is currently in a state of relative stability or gradual decline. Concurrently, the assessment of neutral deviation (Table 3), based on Tajima’s D and Fu’s Fs values at the population level, demonstrated positive and nonsignificant results for both Fu’s Fs (4.792, *P* = 0.006) and Tajima’s D (1.02411, *P* > 0.1). This lack of significant difference (*P* > 0.05) fails to reject the null hypothesis of neutral evolution, implying an absence of recent population expansion events. Additionally, Harpending’s Rag index (HRag) and its Sum of Squared Deviations (SSD) were computed using Arlequin 3.5.2 to assess the smoothness of the mismatch distribution. The results indicated positive and significantly different values for both SSD (0.0313) and HRag (0.0330) (*P* < 0.05), thereby rejecting the hypothesis of population expansion.

**Table 3:** Results of genetic and mismatch analyses in *Rh. Pumilum.* Hrag, Harpending’s rag index; SSD, Sum of Squared deviations

Population Groups	Number of samples	Fu’s Fs, *P*-value	Tajima’s D, *P*-value	SSD, *P*-value	Hrag, *P*-value
All Populations	240	4.792, 0.006	1.02411, > 0.1	0.0313, 0.000	0.0330, 0.000
Group I	130	0.542, 0.117	-1.20911, > 0.1	0.1087, 0.000	0.1074, 0.000
Group II	110	-1.604, 0. 067	-1.49637, > 0.1	0.0497, 0.28	0.0388, 0.24

To further investigate expansion events over the extended evolution of *Rh. pumilum* populations, we categorized 26 populations into two groups based on genetic relationships (referencing Cluster I and II in the UPGMA dendrogram) and conducted separate mismatch distribution analyses. The outcomes revealed double-peak mismatch distribution curves for both Group I (Fig. 5B) and Group II (Fig. 5C), rejecting population expansion hypothesis. Neutrality test results (Table 3) revealed that in Group I, Fu’s Fs presented a non-significant positive value (0.542, *P* = 0.117), and Tajima’s D presented a non-significant negative value (-1.20911, *P* > 0.05), collectively refuting the population expansion hypothesis for *Rh. pumilum*. Conversely, in Group II, Fu’s Fs (-1.604, *P* = 0.067) and Tajima’s D (-1.49637, *P* > 0.05) were both negative, tentatively supporting the expansion hypothesis, although these differences lacked statistical significance (*P* > 0.05). In the mismatch analysis, both groups exhibited positive values for SSD and HRag (Group I: SSD = 0.0497, *P* = 0.0000, HRag = 0.0388, *P* = 0.000; Group II: SSD = 0.0497, *P* = 0.28, HRag = 0.0388, *P* = 0.24). The *P *value in Group I was significant (*P* < 0.05), rejecting the hypothesis of population expansion events. However, the *P* value in Group II was not significant (*P* > 0.05), failing to reject the significant expansion hypothesis. These results suggest that the whole *Rh. pumilum* population has not undergone rapid expansion. However, populations in the northern part of the QTP, such as Qinghai, Gansu and Northwestern Sichuan, have possibly experienced expansion, though not supported by mismatch distribution analysis. Conversely, populations in the Xizang and western Sichuan, have rejected expansion events.

### Estimation of differentiation time for each haplotype branch

Utilizing the divergence time of *Rh. likiangense* and *Rh. sublanceolatum* (10.4–28.8 Ma, 19.6 Ma) [47] and the divergence time of *Rh. likiangense* and the outgroup *Oxyria digyna* (20.8–49.8 Ma, 35.3 Ma) [63] as calibration points for a loose molecular clock, the differentiation time of cpDNA haplotypes in *Rheum* was estimated. The results revealed that the genus *Rheum* diverged from its sister genus *Oxyia* approximately 22.22 Ma (95% HPD: 12.8-32.59 Ma), evolving into numerous species successively (Fig. 4). The earliest origin of *Rheum* species is traced to 14.24 Ma (95% HPD: 6.5-22.11 Ma), followed by *Rheum sublanceolatum* at 13.96 Ma (95% HPD: 7.04–21.12 Ma) (Fig. 4). The most recent common ancestor (MRCA) of *Rheum pumilum* is estimated to have existed around 11 Ma (95% HPD: 5.2-17.13 Ma), corresponding to the middle Miocene of the Tertiary. Subsequent divergence of two major *Rh. pumilum* lineages occurred at approximately 7.09 Ma (95% HPD: 2.53–12.09 Ma) and 6.82 Ma (95% HPD: 2.74–11.38 Ma) during the late Miocene (Fig. 4). These two lineages, which diverged in northeastern Xizang-western Sichuan (lower latitudes) and Qinghai-Gansu-Northwestern Sichuan (higher latitudes), began to separate around the same geological period (Fig. 4). Haplotype differentiation within these two lineages continued until about 0.23 Ma (95% HPD: 0-0.73 Ma). The origin and differentiation of *Rheum* species and *Rh. pumilum* haplotypes can be divided into three major periods: 11–15 Ma, 3.4–7.09 Ma, and 0.23–2.5 Ma. These timeframes align with significant geological epochs, namely the middle Miocene, late Miocene, Pliocene, and Pleistocene (Fig. 4).

### Reconstruction of *Rh. pumilum* distribution changes using ENM

To validate the consistency of the distribution changes simulated by ENM with effective population size changes based on cpDNA population genetics data, 136 distribution sites of *Rh. pumilum* were selected from field surveys and specimen data (http://www.cvh.org.cn, https://www.gbif.org/). These sites span a regional range of 88.7-105.23°E and 27.93–39.57°N. Nineteen climate factor data were extracted from the QTP with a resolution of 2.5 arc-minutes, covering four distinct periods: the LGM at approximately 21 ka, the middle Holocene (Mid-Holocene) at around 6 ka, the current period (1970–2000), and a future projection for 2060–2080. After filtering out climate factors with a correlation greater than 0.8, nine environmental variables were retained (Additional file 2: Table S2). Based on these variables, species distribution model simulation was performed. Results indicated that different environmental factors had varying impacts on the potential distribution of *Rh. pumilum* across different periods. Out of the nine selected environmental factors, Mean Temperature (Bio10) and Precipitation of Wettest Month (Bio13) were found to have the most significant contributions, followed by Temperature Seasonality (Bio4), Max Temperature of Warmest Month (Bio5), and Mean Diurnal Range (Mean of monthly (max temp - min temp)) (Bio2). Altitude, exclusively considered in the current layer, also exhibited a high contribution rate (Additional file 2: Table S2). Therefore, *Rh. pumilum* appeared to be particularly sensitive to factors such as altitude, humidity, and temperature, suggesting a likely association with its tolerance to drought and cold conditions.

**Fig. 6:**
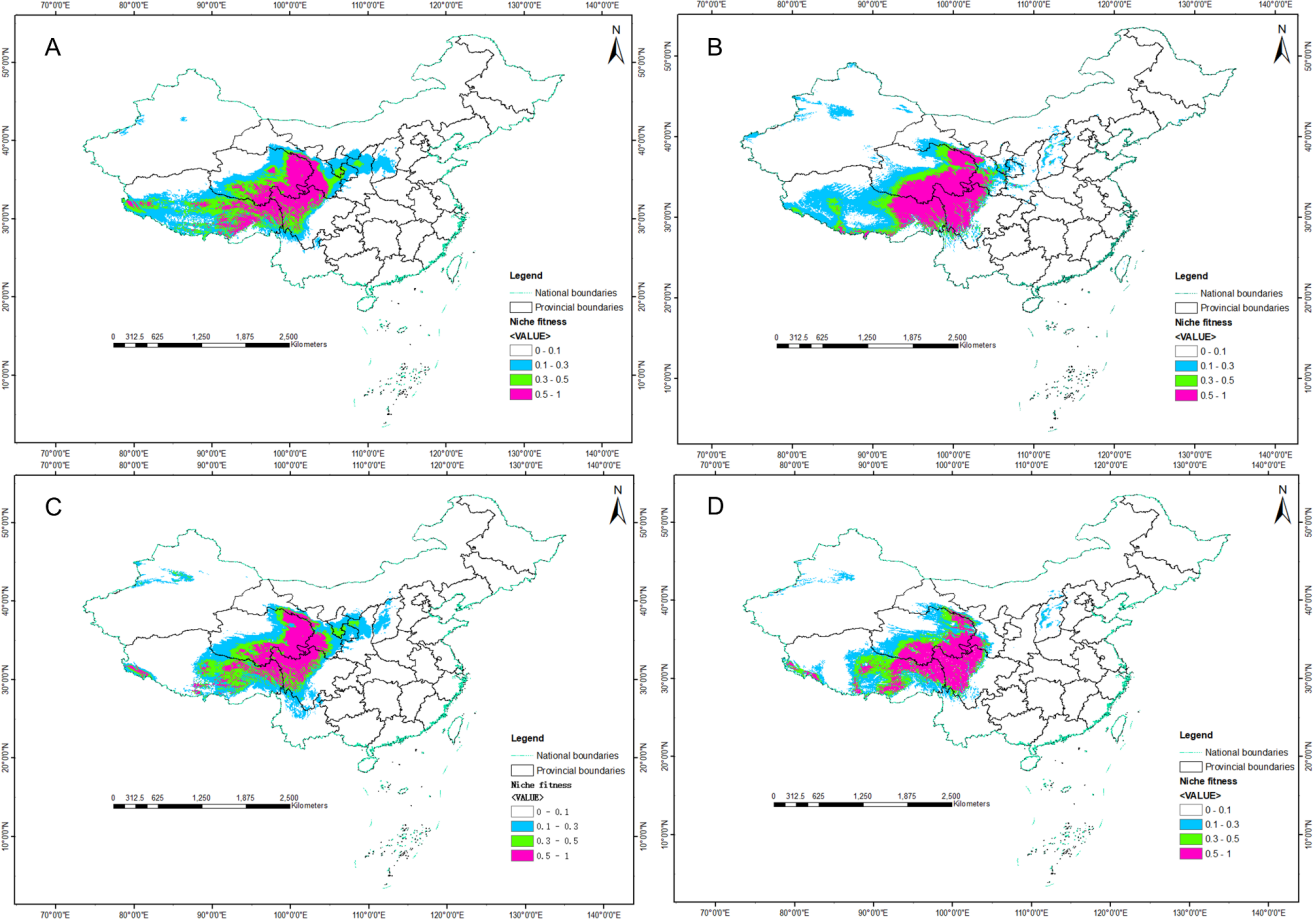
The distribution of potential suitable areas of *Rh. pumilum* in different periods predicted by Ecological Niche Model. **A** The Last Glacial Maximum; **B** The Mid-Holocene; **C** The Current Layer; **D** The Future (2060–2080)

The prediction results for potential suitable areas (Fig. 6, Additional file 5: Table S5) closely aligned with the current distribution of *Rh. pumilum*. Medium and high suitable areas were predominantly situated on the QTP (east and north of Qinghai, northeast of Xizang), Hengduan Mountains in Sichuan, and Qilian Mountains in the northeast of the plateau (Fig. 6). Across the four prediction periods, the total suitable area for *Rh. pumilum* was smallest in the future (2060–2080) (215.97 km^2^) and largest in the Middle Holocene (287.35 km^2^) (Additional file 5: Table S5). Even during the LGM, *Rh. pumilum* exhibited a relatively extensive suitable area (276.56 km^2^), slightly larger than the current suitable area (253.27 km^2^) (Additional file 5: Table S5). The contours of the predicted medium and high suitable areas for the four periods exhibit a general similarity (Fig. 6). The high suitable areas in the current layer have the smallest area (80.21 km^2^), contrasting with a larger extent during the LGM (104.83 km^2^), in addition, the high suitable areas for the Middle Holocene and the future (2060–2080) are 90.26 km^2^ and 86.29 km^2^, respectively (Additional file 2: Table S5). In both the current and future periods, ENM reveals a fragmented distribution pattern for the high suitable areas of *Rh. pumilum* (Fig. 6). Notably, when compared to the LGM, these areas in the Qilian Mountains and Hengduan Mountains exhibit a noteworthy shrinking trend in the last three periods (the Mid-Holocene, the current, the future). This trend might be attributed to glacial retreat and elevation changes in the distribution region of *Rh. pumilum* during interglacial periods.

## Discussion

### Genetic diversity and lineage structure of *Rh. pumilum*

The analysis of genetic diversity based on cpDNA revealed that the total genetic diversity index (Ht) of *Rh. pumilum* was 0.910 (0.0330). This value is comparable to the genetic diversity observed in various reported alpine plants [22, 33, 71]. Notably, the identified genetic diversity surpassed Petit’s average chloroplast variation level, which was 0.673 based on extensive studies of hundreds of plant chloroplasts [43]. Additionally, the total genetic variation observed in *Rh. pumilum* is primarily attributed to between-populations variation rather than within-population variation (Hs = 0.330, F_ST_ = 0.8450). Several factors contribute to the genetic diversity of a species, including evolutionary time, habitat, population size, breeding methods, gene flow, genetic drift, and human activities, etc [38–41, 72, 73]. The high genetic diversity in *Rh. pumilum* can be attributed to multiple interacting factors. Firstly, the long evolutionary history of *Rh. pumilum* has facilitated the accumulation of a broad range of genetic variations. Secondly, historical events, such as the uplift of the QTP and associated climate changes, have increased habitat heterogeneity, promoting adaptive evolution within the species and thereby enhancing genetic diversity. Furthermore, the complex alpine environment of the QTP may restrict gene flow and intensify genetic differentiation between populations, which is supported by gene flow analysis (Nm = 0.04). In small-scale *Rh. pumilum* populations, genetic drift—such as bottleneck and founder effects—may lead to the fixation of certain genotypes and the loss of others, thus reducing within-population genetic diversity (Hs = 0.330). However, selection pressures and founder effects during population expansion may also contribute to the genetic diversity between populations. The combined influence of these factors has resulted in the high genetic diversity observed in *Rh. pumilum* populations. In this study, a joint analysis of three gene fragments was conducted across 240 individuals in 26 populations, obtaining a total of 39 haplotypes. The distribution of haplotypes in *Rh. pumilum* reveals a genetic differentiation pattern characterized by the coexistence of multiple dominant haplotypes occurring in various populations (Fig. 1; Table 2). These dominant haplotypes exhibit distinct regional distribution characteristics. Moreover, a prevalence of unique haplotypes in the *Rh. pumilum* populations also has been found. It is hypothesized that different ecological niches of alpine habitat *Rh. pumilum populations* may have favored the fixation of specific haplotypes, resulting in a genealogical distribution pattern similar to “alpine-island” [33].

The genetic analysis conducted on *Rh. pumilum* populations reveals the relationship between genetic and geographical distances. The genetic differentiation coefficients, N_ST_ (0.897) and G_ST_ (0.638), provide valuable information. The significantly greater N_ST_ compared to G_ST_ implies a positive correlation between genetic and geographical distance in *Rh. pumilum.* This indicates a pronounced phylogeographic structure within the distribution of *Rh. pumilum*. The UPGMA dendrogram based on genetic distances, PCoA analysis, and Structure analysis collectively support the observed phylogeographic (Fig. 2). Populations with closer geographical proximity cluster together, illustrating a clear “north-south” distribution pattern based on latitude. For instance, populations from Xizang and western Sichuan form one cluster, while those from Qinghai, Gansu, and Northwestern Sichuan form another. This pattern is further supported by the phylogeny tree based on 39 haplotypes of *Rh. pumilum*, which displays two major lineage branches corresponding to the two distribution regions, demonstrates that *Rh. pumilum* has two relatively independent phylogeographic histories. The Mantel test, analyzing the correlation between average genetic distance and geographical distance, yields a strong and statistically significant positive correlation (*r* = 0.5522, *P* < 0.01). This result suggests that the distribution of *Rh. pumilum* populations adheres to a geographical isolation model, supporting the concept of geographical isolation as a driving force in population dynamics. The influences of geographical isolation are profound. Such isolation can lead to the fixation or loss of alleles within local populations, intensifying genetic drift effects and consequently promoting population differentiation [74, 75]. This comprehensive analysis contributes to our understanding of the intricate interplay between genetic diversity and geographical factors in shaping the population structure of *Rh. pumilum*.

### Estimation of the maternal origin and branch differentiation time of *Rh. pumilum*

Previous studies have found that there may be allopolyploidy in the origin *Rh. pumilum* species, but the origin of its parents has not been determined [45, 47]. The phylogeny tree constructed based on cpDNA (Fig. 4) revealed a closer relationship between *Rh. pumilum* and *Rh. sublanceolatum* with a robust support rate of 100%, and another noteworthy observation is the closer relationship of *Rh. kialense* with haplotypes H14 and H29, with the support rates of 99%. This aligns with earlier findings based on phylogeny tree of *Rheum* species by both chloroplast and nucleic gene fragments, *Rh. pumilum* clustered with *Rh. kialense* and *Rh. sublanceolatum*, boasting high support rates (ITS: BP = 94%, BS = 60%; cpDNA: BP = 100%, BS = 100%) [45, 47]. Given that chloroplast genes are maternally inherited in this genus and considering the existence of tetraploid individuals in *Rh. pumilum*, these relationships may imply *Rh. kialense*’s potential involvement as a maternal parent in the polyploid origin of *Rh. pumilum*. The common distribution of the two species in northern Sichuan and southern Gansu also offers possibilities. Similarly, *Rh. sublanceolatum* may have been involved in the origin of *Rh. pumilum* species. However, it is essential to underscore that the conclusive affirmation necessitates further data support.

The estimation of the haplotype differentiation time for *Rh. pumilum* was conducted using BEAST software, revealing that the genus *Rheum* began diverging around 22.22 Ma during the middle Miocene (Fig. 4). This finding is consistent with earlier studies suggesting that *Rheum* diverged from its sister genus *Oxyria* approximately 20.8 Ma (17.9–22.6 Ma) based on cpDNA [47]. Although these estimates require further testing with additional robust phylogenies and more reliable fossil evidence, our study and previous research converge on the rapid radiation of *Rheum* between 9.9 and 14 Ma during the middle Miocene [47]. These results align with the observed radiative diversification of many genera and species on the QTP during the period, likely driven by significant uplifts and intensified monsoon climates [76–78] that altered temperature and humidity patterns, which are believed to have catalyzed explosive diversification in these species. Additionally, the speciation time of *Rh. pumilum* is estimated at approximately 11 Ma (5.2-17.13 Ma), in the interspecific differentiation peaking of *Rheum* between 9.9 and 14 Ma [47], reflecting the influence of the uplift of the QTP and middle Miocene monsoon climate. Subsequent peaks of intraspecific differentiation occurred between 3.4 and 7.09 Ma and 0.23–2.5 Ma likely linked to the uplift of the QTP during the late Miocene-Pliocene [79] and climatic changes during the Pleistocene-Holocene periods [80, 81]. These geological and climatic shifts led to habitat fragmentation, isolation, and intraspecific lineage differentiation, with the QTP uplift and Quaternary climate fluctuations playing key roles in the differentiation of *Rh. pumilum* [82].

### Ice age refugia and historical dynamics of *Rh. pumilum*

The high genetic and haplotype diversity within *Rh. pumilum* populations distributed across the Hengduan Mountains in Sichuan, the plateau edge in Gansu, and the platform of the QTP (Table 2), which suggests that both the plateau edge and plateau platform may have acted as refugia for *Rh. pumilum* during the glacial period, despite the lack of evidence for ancestral haplotypes. In contrast, other populations with low levels of haplotype and genetic diversity may have expanded after the glacial period, having undergone the founder effect and genetic drift effects during their establishment. This aligns with the second model of plant refuge on the QTP, which identifies that multiple refugia exist on both the plateau platform and edge during the LGM [12, 22–26]. These results suggest micro-refugia existed on the plateau platform, opposing the first model which suggests refugia only at low altitudes. This challenges the idea that large ice sheets on the QTP during the LGM caused extinction of all plant species [83, 84], a view refuted by studies on alpine plants [19, 20].

Mismatch distribution analysis showed no population expansion in *Rh. pumilum*, indicating a recently stable or slowly declining population. Genetic data revealed a significant correlation between population structure and geographical distance, with a clear “south-north” latitudinal distribution. Haplotype network analysis further supports this, as the population network formed a long-line shape rather than a “star-shape”, suggesting no rapid expansion.

Using the MaxEnt model, four historical suitable areas for *Rh. pumilum* were simulated. Results showed that temperature and humidity were critical factors shaping its historical distribution, with the highest suitability found in regions such as the QTP platform, Hengduan Mountains, and the Qilian Mountains (Fig. 6, Additional file 5: Table S5). Comparing suitability across four time periods, the LGM period had the largest suitable area, followed by the Middle Holocene and the future for 2060–2080, with the current distribution being the smallest (Fig. 6, Additional file 5: Table S5). This supports the idea that alpine plants like *Rh. pumilum* migrated from high to lower altitudes during the LGM, leading to expanded and interconnected distribution areas [13, 15–17]. However, during the Holocene, its distribution has contracted and fragmented, likely due to altitude and environmental changes alterations in monsoon climate patterns, and rises in temperature and humidity. As glaciers retreated, populations moved to higher altitudes, increasing environmental heterogeneity, habitat fragmentation. Additionally, in the current layer, human activity and the ongoing greenhouse effect, causing higher temperatures and humidity change, may further restrict *Rh. pumilum*’s distribution. In the future for 2060–2080, the most suitable area for *Rh. pumilum* has increased slightly compared to the current layer, and it is speculated that the reason may be the control of human activities. Unlike the typical glacial retreat and interglacial expansion observed in many species [30–33], *Rh. pumilum* follows a “displacement refugia” model, similar to European plateau plants [29]. This suggests that during the LGM, *Rh. pumilum* did not shrink but rather expanded due to lower habitat elevations, facilitating population connectivity. However, as habitats became fragmented during interglacial periods, populations became isolated, leading to contraction. Genetic analyses confirm significant population differentiation and low gene flow, reinforcing the role of habitat heterogeneity and climate oscillations in shaping the genetic structure of *Rh. pumilum*. These changes might be strongly influenced by the uplift of the QTP and climatic shifts during the Quaternary period.

## Conclusion

This study presents a comprehensive molecular phylogeographic analysis of the endemic species *Rh. pumilum* on the QTP. Through an in-depth investigation of chloroplast gene segments from 26 *Rh. pumilum* populations, we have unveiled the genetic characteristics and evolutionary history of this species on the QTP. The study elucidates a phylogeographic distribution pattern akin to the “alpine-island” model. *Rh. pumilum* demonstrates a notable high level of genetic diversity and a significant phylogeographic structure, emphasizing the pivotal role of geographical isolation in its evolutionary process. The integration of ENM and genetic analysis highlights the high sensitivity of *Rh. pumilum* to environmental factors, including altitude, temperature, and humidity, illustrating its habitat shifts across different periods. Furthermore, the phylogenetic analysis involving closely related species of *Rh. pumilum* provides insights into potential polyploidy origins. These findings significantly contribute to our understanding of the evolutionary history of plants on the QTP, underscoring the dominant influence of geological history events on the evolution of *Rh. pumilum*, and providing substantial theoretical groundwork for biodiversity conservation.

## Electronic supplementary material

Below is the link to the electronic supplementary material.

Supplementary Material 1

Supplementary Material 2

Supplementary Material 3

## Data Availability

All used sequencing data are available from the GenBank with the accession numbers: PP049090 - PP049128 for trnL-F sequences, PP049129 - PP049167 for matK sequences, PP049168 - PP049206 for trnS-G sequences Sequence.
