# Supplementary figures and images for: Genetic differentiation and historical dynamics of the endemic species Rheum pumilum on the Qinghai-Tibetan Plateau inferred from phylogeography implications
Source: BMC Plant Biol. 2025 Feb 7;25:162. doi: 10.1186/s12870-025-06164-y (PMC11803965; doi:10.1186/s12870-025-06164-y)

$$\text{DeltaK} = \text{mean}(|L''(K)|) / \text{sd}(L(K))$$

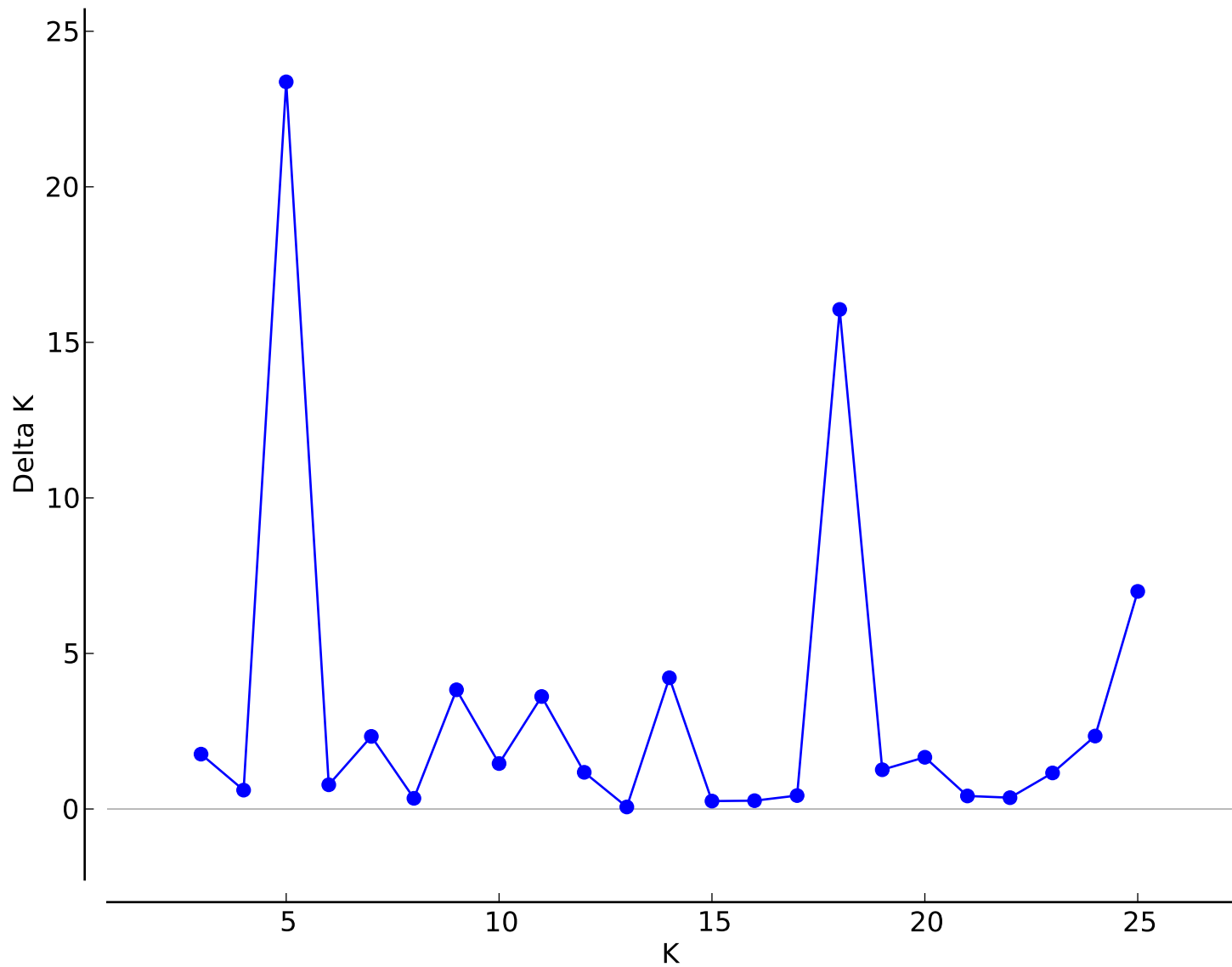

Supplement: Supplementary file 1 — Supplementary Material 1 [file 12870_2025_6164_MOESM1_ESM.pdf]
