# Supplementary material for: Genetic differentiation and historical dynamics of the endemic species Rheum pumilum on the Qinghai-Tibetan Plateau inferred from phylogeography implications
Source: BMC Plant Biol. 2025 Feb 7;25:162. doi: 10.1186/s12870-025-06164-y (PMC11803965; doi:10.1186/s12870-025-06164-y)
Supplement: Supplementary file 2 — Supplementary Material 2 [file 12870_2025_6164_MOESM2_ESM.docx]

Table S1. The species used in BEAST analysis and accession numbers of the *mat*K, *trn*L-F and *trn*S-G gene segments.

Table S2. The environmental variables used in the niche modeling.

Table S3. The variation sites of haplotypes of *Rh. pumilum.* Different Capital letters represent different nucleic acid sequences: B=CGTTATTATAAAAATTC, D=TAAA, E=ATATATAA, F=AACTATATATTAT, H=ATGCCCTATTGATTATCCTTTCTTTT, I=TTAAA, J=AA，K=TT, L=TTAATA，M=TTTT，N=TTATA, O=TTAT，P=TA，Q=ATAATAA，R=TATAATAA, S=TTTAT, U=ATAT, V=AT, W=AATAAT, X=TTATGT, Y=TTTTT, Z=TTTATAACTCTATTAATACTTT.

Table S4. Structure of genetic variation within and between populations of *Rh. pumilum.* F_ST_, differentiation among populations within the species.

Table S5. The potential suitable areas for *Rh. pumilum* populations predicted by Ecological Niche Modeling.

Figure S1. Estimated values of Deltak (ΔK) on structure analysis
